# Complete genome sequence and analysis of *Lactobacillus hokkaidonensis* LOOC260^T^, a psychrotrophic lactic acid bacterium isolated from silage

**DOI:** 10.1186/s12864-015-1435-2

**Published:** 2015-03-25

**Authors:** Yasuhiro Tanizawa, Masanori Tohno, Eli Kaminuma, Yasukazu Nakamura, Masanori Arita

**Affiliations:** Department of Computational Biology, Graduate School of Frontier Sciences, The University of Tokyo, Chiba, 277-8561 Japan; Center for Information Biology, National Institute of Genetics, Shizuoka, 411-8540 Japan; National Agriculture and Food Research Organization, National Institute of Livestock and Grassland Science, Tochigi, 329-2793 Japan; RIKEN Center for Sustainable Resource Science, Kanagawa, 230-0045 Japan

**Keywords:** Lactic acid bacteria, Silage fermentation, Cold adaptation, Pentose metabolism, Mobile genetic element, Integrative and conjugative element

## Abstract

**Background:**

*Lactobacillus hokkaidonensis* is an obligate heterofermentative lactic acid bacterium, which is isolated from Timothy grass silage in Hokkaido, a subarctic region of Japan. This bacterium is expected to be useful as a silage starter culture in cold regions because of its remarkable psychrotolerance; it can grow at temperatures as low as 4°C. To elucidate its genetic background, particularly in relation to the source of psychrotolerance, we constructed the complete genome sequence of *L. hokkaidonensis* LOOC260^T^ using PacBio single-molecule real-time sequencing technology.

**Results:**

The genome of LOOC260^T^ comprises one circular chromosome (2.28 Mbp) and two circular plasmids: pLOOC260-1 (81.6 kbp) and pLOOC260-2 (41.0 kbp). We identified diverse mobile genetic elements, such as prophages, integrated and conjugative elements, and conjugative plasmids, which may reflect adaptation to plant-associated niches. Comparative genome analysis also detected unique genomic features, such as genes involved in pentose assimilation and NADPH generation.

**Conclusions:**

This is the first complete genome in the *L. vaccinostercus* group, which is poorly characterized, so the genomic information obtained in this study provides insight into the genetics and evolution of this group. We also found several factors that may contribute to the ability of *L. hokkaidonensis* to grow at cold temperatures. The results of this study will facilitate further investigation for the cold-tolerance mechanism of *L. hokkaidonensis*.

**Electronic supplementary material:**

The online version of this article (doi:10.1186/s12864-015-1435-2) contains supplementary material, which is available to authorized users.

## Background

Silage fermentation is promoted mainly by the microbial activities of lactic acid bacteria (LAB). During the fermentation process, LAB produce lactic acid anaerobically as the major end product of central carbohydrate metabolism, which reduces the pH of the surrounding environment. These anaerobic and acidic conditions prevent the propagation of detrimental microorganisms such as listeria, clostridia, yeasts, and other fungi. However, the acid production level tends to be insufficient if silage is prepared in cold weather conditions because of the impaired activity of LAB, thereby yielding lower quality silage. Therefore, the inoculation of appropriate LAB as a silage additive is required to enhance silage fermentation in low-temperature environments.

Previously, we isolated a novel psychrotrophic *Lactobacillu*s species, *Lactobacillus hokkaidonensis*, from Timothy grass (*Phleum pratense*) silage in Hokkaido, a subarctic region of Japan [1]. *L. hokkaidonensis* can grow at temperatures as low as 4°C (optimal growth at 25°C), and its type strain LOOC260^T^ was shown to decrease pH even in cold conditions when used to inoculate pilot-scale grass silage. Thus, *L. hokkaidonensis* is expected to be suitable for use as an effective silage inoculant in cold regions.

*L. hokkaidonensis* is classified as an obligate heterofermentative LAB in the *L. vaccinostercus* group [2], which includes five species (*L. vaccinostercus* [3], *L. suebicus* [4], *L. oligofermentans* [5], *L. nenjiangensis* [6], and *L. hokkaidonensis*) that form a clade distinct from the well-known heterofermentative clades, which include *L. reuteri*, *L. brevis*, and *L. buchneri*. They share common phenotypic features such as the presence of *meso*-diaminopimelic acid in their peptidoglycan cell walls and faster assimilation of pentoses compared with hexoses, but little is known about their genetic background or genomic information.

In the present study, we performed whole-genome sequencing of *L. hokkaidonensis* LOOC260^T^ and comparative genome analysis, where we focused on the unique gene repertoire of the *L. vaccinostercus* group. In addition, determining the complete genome may provide a better genome-wide understanding of mobile genetic elements, thereby highlighting how flexible genome rearrangements contribute to adaptation to various ecological niches. Thus, we aimed to provide insights into the genomic features of the *L. vaccinostercus* group, which is poorly characterized at present, as well as to clarify the silage fermentation mechanism from a genomic perspective, particularly in cold conditions.

## Results and discussion

### Genome features of *L. hokkaidonensis* LOOC260^T^

**Figure 1:**
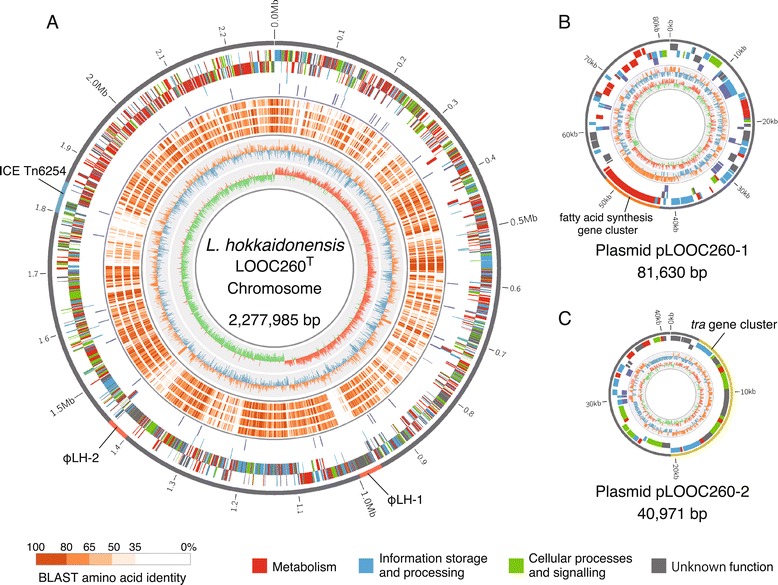
**Genome atlas of** ***L. hokkaidonensis*** **LOOC260** ^**T**^ **. A)** Chromosome. The outer four circles (from outer to inner) represent CDSs on the forward strand, CDSs on the reverse strand, rRNAs (red) and tRNAs (blue), and insertion sequences/transposases, respectively. The next four circles (from outer to inner) represent the shared amino acid identities of the BLAST alignments with four closely related species: *L. oligofermentans* DSM 15707^T^, *L. vaccinostercus* DSM 20634^T^, *L. vaccinostercus* DSM 15802, and *L. suebicus* KCTC 3549^T^, respectively. The inner two circles represent the GC content and GC skew. **B**, **C)** Plasmids pLOOC260-1 and pLOOC260-2. From the inner to outer circles: CDSs on the forward strand, CDSs on the reverse strand, insertion sequences/transposases, GC content and GC skew. The CDSs are colored according to the main COGs functional classification categories: red, metabolism; blue, information storage and processing; red, cellular processes and signaling; gray, unknown function.

Whole-genome sequencing was conducted with the PacBio single-molecule real-time (SMRT) sequencing system to determine the genome sequence of *L. hokkaidonensis* LOOC260^T^. *De novo* assembly using the hierarchical genome assembly process (HGAP) method [7] generated seven contigs, which were further assembled and verified to finish the single complete genome. The genome of LOOC260^T^ comprises one circular chromosome (2,277,985 bp) and two circular plasmids designated as pLOOC260-1 (81,630 bp) and pLOOC260-2 (40,971 bp). Two prophage regions were predicted, which are described in detail in the following section. No clustered regularly interspaced short palindromic repeat (CRISPR) loci were detected in the genome. The general genomic features of *L. hokkaidonensis* LOOC260^T^ and four other species in the *L. vaccinostercus* group are summarized in Table 1. Figure 1 shows the genome atlas of LOOC260^T^ as well as BLASTP alignment results with its four close relatives, as described above. Sharp transitions in the GC-skew value were observed at both the predicted *ori*C site (0°) and its opposite site (176°). In particular, genes involved in metabolism (indicated in red) were densely encoded in the region from 300° to 360°. Several genes in this region were missing from all or some of the members of the *L. vaccinostercus* group, which may reflect the adaptation to specific ecological niches during the diversification of this group. Similar position-specific features have also been reported in *L. plantarum* [8] and *L. casei* [9], where they are considered to be lifestyle adaptation islands.

**Table 1:** **Genome features of** ***L. hokkaidonensis*** **LOOC260** ^**T**^ **and** ***L. vaccinostercus*** **group species**

**Strain**	**Status**	**No. of sequences**	**Total bases**	**% GC**	**CDSs**	**rRNA operons**	**tRNAs**	**INSD/SRA accession no.**
*L. hokkaidonensis* LOOC260^T^ (Timothy grass silage)	Complete	3	2,277,985	38.2	2,194	4	56	AP014680^#^
	(1 Chromosome +2 Plasmids)		81,630	40.4	99	0	0	AP014681^#^
			40,971	39.4	51	0	0	AP014682^#^
*L. oligofermentans* DSM 15707^T^ (Modified atmosphere-packaged poultry products)	Scaffold	16	1,789,770	35.5	1,742	-	52	SRR1151187**
*L. vaccinostercus* DSM 20634^T^ (Cow dung)	Scaffold	88	2,551,457	43.5	2,471	-	52	SRR1151143**
*L. vaccinostercus* DSM 15802* [Acid-fermented condiment (tempoyak) in Malaysia]	Scaffold	129	2,558,791	43.5	2,506	-	53	ERR387466**
*L. suebicus* KCTC 3549^T^ (Apple mash)	Scaffold	143	2,656,936	39.0	2,583	-	55	BACO01000000

^#^This study. *Formerly named *L. durianis*. **SRA accession no.

### Diverse mobile genetic elements harbored by *L. hokkaidonensis* LOOC260^T^

Bacterial genomes include several repetitive sequences such as multiple copies of ribosomal RNA operons and insertion sequences or transposases. These regions are generally difficult to reconstruct from relatively short sequencing reads, and thus *de novo* assembly often yields collapsed and/or fragmented contigs for such regions. We used the PacBio sequencer to correctly assemble these repetitive regions with much longer reads (4 kbp on average), thereby obtaining a genome-wide perspective of mobile genetic elements such as plasmids and prophages.

#### Insertion sequences

In total, 59 ORFs, including partial ORFs and pseudogenes, were annotated as putative insertion sequences within the genome. In particular, three types of insertion sequence elements were annotated, with 13, 6, and 3 copies that shared almost 100% identity, and these new insertion sequence elements were registered in the ISfinder database [10] as ISLho1, ISLho2, and ISLho3, respectively. They shared 66% amino acid similarity with ISLre2 (*L. reuteri*), 75% with ISLrh2 (*L. rhamnosus*), and 60% with ISLre1 (*L. reuteri*), respectively.

#### Plasmids

The ratio of the mapped read number normalized against the sequence length for each replicon was approximately 1:1:4 (chromosome:pLOOC260-1:pLOOC260-2). Thus, the plasmid copy number in the cell was estimated as one for pLOOC260-1 and multiple for pLOOC260-2.

The first plasmid, pLOOC260-1, had a composite structure that comprised regions from several LAB species, such as *L. plantarum, L. casei, L. brevis*, and *L. coryniformis*, thereby indicating the occurrence of numerous rearrangements and recombination events during its evolution. The plasmid mobilization protein, Mob, gene was present, which probably facilitated the transmission of pLOOC260-1 in the presence of other conjugation mechanisms. Another interesting characteristic was the presence of a gene cluster related to fatty acid synthesis (LOOC260_200520–LOOC260_200630), which was absent from the chromosome. To the best of our knowledge, plasmid-encoded fatty acid synthesis genes have not been reported previously in other LAB species.

The other plasmid, pLOOC260-2, was considered to be a conjugative plasmid. It possessed a *tra* conjugation gene cluster, which shared high similarity and colinearity with the plasmid pWCFS103 from *L. plantarum* WCFS1, for which conjugative transfer was demonstrated experimentally [11]. A similar gene organization in the *tra* region is also observed in several plant-associated LAB, such as *L. brevis* KB290, isolated from a Japanese fermented vegetable [12], *L. oryzae*, isolated from fermented rice grains [13], and *L. coryniformis*, frequently isolated from silage.

#### Prophages

**Figure 2:**
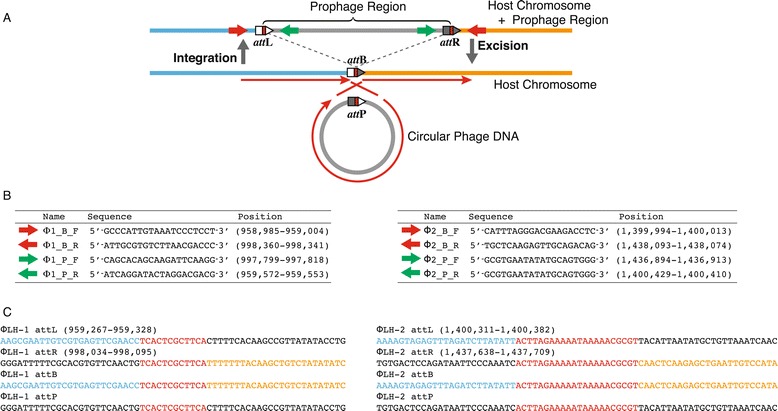
**Phage attachment sites. A)** Schematic representation of the integration and excision of prophages. *att*P, *att*B, *att*L, and *att*R represent the phage attachment sites. Inward red arrows and outward green arrows indicate the PCR primers designed to amplify the *att*B and *att*P regions, respectively. **B)** Primer sequences for φLH-1 and φLH-2. **C)** Nucleotide sequences of the attachment sites for φLH-1 and φLH-2. Red letters represent the core sequences of the phage attachment sites. Blue and orange letters correspond to the left and right sequences of the prophage regions.

Two prophage loci were predicted in the chromosome, φLH-1 (959–998 kb) and φLH-2 (1,400–1,437 kb). We also found 12-bp direct repeats (5′-TCACTCGCTTCA-3′) flanking φLH-1 and 22-bp direct repeats (5′-ACTTAGAAAAATAAAAACGCGT-3′) flanking φLH-2, which appeared to constitute the core regions of phage attachment sites (*att*R and *att*L). A contig obtained by *de novo* assembly contained a misassembled region, which was presumably derived from an excised circular phage DNA, and thus spontaneous excision of the prophage must have occurred in a fraction of the cells. To confirm this prediction by PCR, two sets of primers were designed for each prophage so the fragments could be amplified only when the prophages were excised from the chromosome (Figure 2A,B). The expected PCR products were obtained, and the direct repeats located at the phage attachment sites were identified by sequencing the amplicons (Figure 2C). In the *L. vaccinostercus* group, these prophages are the first instances whose sequences have been determined and whose excision has been demonstrated.

#### Integrated and conjugative elements

Integrated and conjugative elements (ICEs), sometimes known as conjugative transposons, are self-transmissible mobile genetic elements, which can be integrated into or excised from the host chromosome [14]. ICEs often contain accessory genes that confer advantages on their hosts, such as resistance to antibiotics, heavy metals, or phages [15]. ICEs have been reported frequently in *Streptococci* and *Enterococci*, and they are well characterized, but only two previous studies have described ICEs in the genus *Lactobacillus*: one in *L. paracasei* [16] and the other in *L. salivarius* [17]. No other ICEs are registered in the two ICEs/transposable elements databases: ICEberg [18] and Tn Number Registry [19].

We identified a putative ICE in the genome of LOOC260^T^ in the chromosome region 1,799–1,851 kbp (approximately 52 kbp), which was deposited as Tn6254 in the Tn Number Registry.

**Figure 3:**
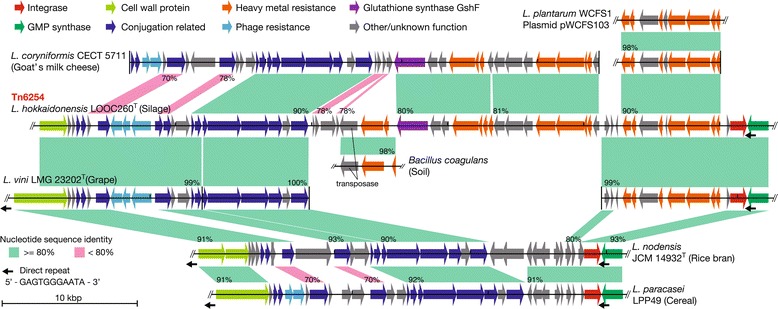
**Comparisons of integrated and conjugative elements from** ***L. hokkaidonensis*** **and several species in the genus** ***Lactobacillus.*** Green and red correspond to the nucleotide identity based on BLASTN alignments and the numbers indicate the identity. Small black arrows represent direct repeat sequences flanking the element.

The amino acid sequences of the genes in the ICE region of LOOC260^T^ were compared against all the sequences in the NCBI non-redundant protein database, and similar gene organizations with high levels of sequence identity (>90%) were found in four species of plant-related LAB: *L. vini* LMG 23202^T^ (isolated from grape must), *L. nodensis* JCM 14932^T^ (from rice bran), *L. paracasei* LPP49 (from cereal), and *L. coryniformis* (from cheese, silage, and kimchi). The level of shared nucleotide identity was also high between them (Figure 3). In particular, the 20-kbp upstream and 11-kbp downstream segments of Tn6254 were almost identical to the putative ICE from *L. vini* LMG 23202^T^, but Tn6254 had more accessory genes, especially for heavy metal resistance, in the middle 21-kbp region. The integrase genes were adjacent to the 3′-end of the GMP-synthase gene, and direct repeats of 5′-GAGTGGGAATA-3′ were identified at both the 3′-end of the GMP-synthase gene and the 5′-end of the cell wall protein gene. The 3′-end of the GMP-synthase gene is reported to be an integration hotspot for genomic islands, and the consensus sequence of the direct repeats agreed with our findings [20]. However, in LOOC260^T^, we found the same repeat sequence only at the integrase end and not at the opposite end because of the truncated 5′-end of the cell wall protein gene. Therefore, Tn6254 may no longer be capable of excision.

The shared sequence identities were high only within the strains described above. In particular, the four integrase genes shown in Figure 3 shared over 96% amino acid identity, whereas they exhibited lower identities (≤60%) with other known integrase genes. This suggests that these ICEs compose a single family and integrate themselves into the downstream region of the GMP-synthase. Heavy metal resistance genes are beneficial for plant-associated bacteria due to the fact that plants are exposed to metals in the soil, and may even absorb them. However, given their distinct ecological niches, it is unlikely that these ICEs were transferred directly between each strain. This suggests the existence of a large shared gene pool among plant-associated LAB.

### Cold adaptation strategy

Cells exposed to low temperatures undergo significant physiological changes, such as decreases in membrane fluidity and stabilization of the secondary structures of nucleic acids, thereby resulting in less efficient transcription and translation [21]. In bacterial cell membranes, cold temperature induces fatty acid profile changes, such as the conversion of saturated fatty acids into unsaturated fatty acids and the preferential synthesis of short-chain, branched-chain, and/or anteiso fatty acids [22]. However, we found no distinctive characteristics related to the modification of fatty acid composition; we identified no genes involved in the synthesis of unusual fatty acids, such as unsaturated or branched-chain fatty acids, and we found that the number and order of the genes in the fatty acid biosynthesis gene cluster were identical to those in other species, except that they were encoded in the plasmid and not in the chromosome. Low temperatures also induce the production of several proteins such as cold shock protein A (CspA), which functions as an RNA chaperone, and RNA helicase DeaD, which prevents the formation of structured nucleic acids [23]. However, the numbers of these proteins differed slightly from those in the other 17 LAB strains included in the comparative analysis.

The cold stress response is also associated with different types of anti-stress mechanisms. Compatible solutes are chemical compounds, such as betaine and carnitine, that act as osmolytes and confer osmotic tolerance. They also facilitate psychrotolerance, although this physiological mechanism still needs clarification [24]. The uptake and accumulation of compatible solutes in a cold-stressed environment, and the contributions of these solutes to psychrotolerance have been reported in several microorganisms, including *Listeria monocytogenes*, *Yersinia enterocolitica*, and *Bacillus subtilis* [24-26]. In *L. hokkaidonensis*, we found four transporters that were probably responsible for the uptake of these osmolytes: one BCCT family transporter (LOOC260_121750) and three ABC transporters (LOOC260_103390–103400, LOOC260_110220–110250, and LOOC260_117540–117560). The gene repertoire of these transporters was identical to that of *L. sakei*, a psychrotrophic LAB, in which the accumulation of compatible solutes is considered to be a key factor during acclimation to cold and saline environments [27]. Another notable feature was a bifunctional glutathione synthase encoded in the ICE region, GshF (LOOC260_118620), which allows glutathione to be synthesized via two-step ligation from its constituent amino acids [28]. Two key genes involved in the redox cycle of glutathione were also encoded: glutathione peroxidase (LOOC260_117530) and glutathione reductase (LOOC260_103410). Glutathione, which maintains cell redox homeostasis, also protects membrane lipids from the oxidative stress induced at cold temperatures [29]. In *L. hokkaidonensis*, GshF shared high similarity with that in *L. coryniformis*, which was a predominant isolate when we screened for psychrotolerant LAB in Timothy grass silage (see Additional file 1: Figure S1), thereby indicating that glutathione may facilitate psychrotolerance in both species*.*

Bacterial defense systems that protect against cold environments involve a wide range of proteins, including those related to modifications of cell membrane lipids, transcription and translation mechanisms, and various stress proteins [22,23]; therefore, it is difficult to elucidate their direct evidence solely from the viewpoint of genomics. Hence, we will be conducting further investigations, including an expression study using whole-transcriptome sequencing (RNA-seq).

### Unique gene repertoire of the *L. vaccinostercus* group

**Figure 4:**
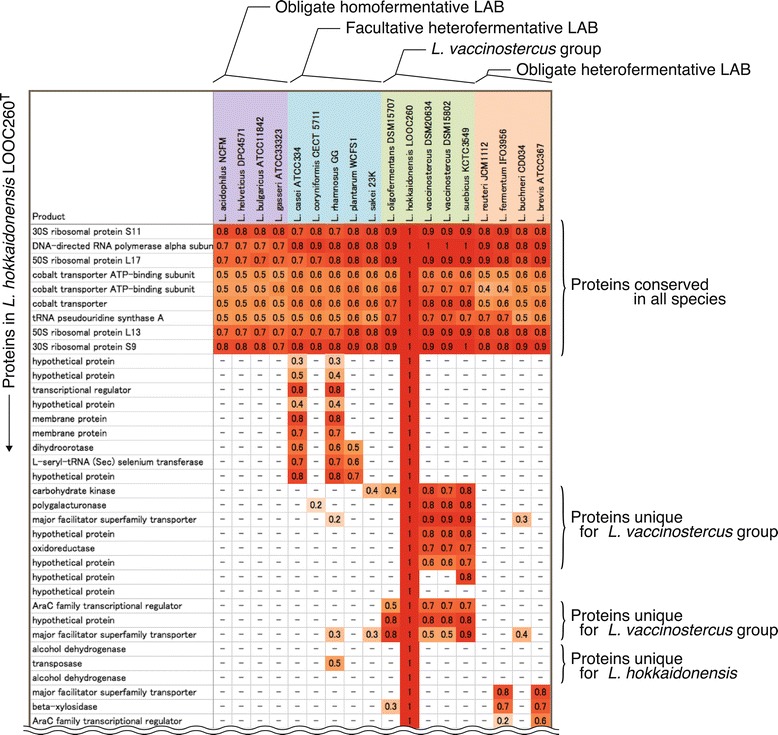
**Ortholog table.** Constructed based on the all-against-all BLASTP alignments between each two species. In the vertical direction, the proteins are shown in order of appearance in the genome of *L. hokkaidonensis* LOOC260^T^. In the horizontal direction, the species included in the comparison are shown. For each row, the bit scores were normalized by dividing by the maximum value. The number of each cell represents the normalized score, and the cells are colored varying shades of red, according to their values, with a deeper color corresponding to a higher value.

To clarify the characteristic gene features of *L. hokkaidonensis* and its close relatives, a comparative analysis was performed using four strains in the *L. vaccinostercus* group and 13 strains from representative LAB species. We generated all-against-all bidirectional BLASTP alignments between *L. hokkaidonensis* LOOC260^T^ and each reference strain. An ortholog table was constructed based on the alignment results. Figure 4 shows some of the table, and Additional file 2 contains the whole dataset. The phylogenetic tree of the 17 strains included in the analysis and the summarized metabolic pathway prediction results obtained using the KEGG Automatic Annotation Server (KAAS) are shown in Additional file 1: Figure S2 and in Additional file 3, respectively.

#### Central metabolism

Similar to the well-characterized heterofermentative LAB, *L. buchneri* [30], all four species in the *L. vaccinostercus* group possessed phosphoketolase, a key enzyme in heterolactic fermentation, but they lacked two genes involved in the Embden–Meyerhof pathway: phosphofructokinase-1 and fructose-bisphosphate aldolase. This was consistent with their classification as obligate heterofermentative LAB. They also possessed two key genes involved in the nonoxidative branch of the pentose phosphate pathway: transketolase and transaldolase. Both L- and D-lactate dehydrogenase were encoded, which agrees with the phenotypic trait that both L-lactate and D-lactate are produced. In contrast to many of the obligate heterofermentative LAB, they lacked genes involved in the arginine deiminase pathway, which differentiates this group from the relatively closely related *L. reuteri* group. The reconstructed carbohydrate metabolism pathway is shown in Additional file 1: Figure S3.

The species in the *L. vaccinostercus* group can assimilate pentoses, such as L-arabinose, D-ribose, and D-xylose, more rapidly than D-glucose, thereby indicating a preference for pentoses over hexoses [1,5,31]. The weak capacity for glucose utilization may be attributed to the cellular redox imbalance caused by insufficient regeneration of NAD(P)^+^ because the *L. vaccinostercus* growth rate on glucose is accelerated by adding electron acceptors, such as aldehydes and ketones, to the medium [31,32]. These characteristics are similar to *Fructobacillus* species, which lack the *adhE* (bifunctional alcohol/acetaldehyde dehydrogenase) gene for regenerating NAD(P)^+^ in the latter stage of heterolactic fermentation [33]. By contrast, members of the *L. vaccinostercus* group possess *adhE*, which suggests that another mechanism is active.

As a starter culture for silage fermentation, the ability to assimilate pentoses is advantageous when utilizing substrates derived from plant cell walls. Hemicellulose is one of the major components of the plant cell wall, which is composed of a branched heteropolymer of saccharides [34]. During the ensiling process, hemicellulose is partially hydrolyzed to yield pentoses, such as xylose and arabinose, which are then fermented into lactic and acetic acid via the phosphoketolase pathway [35]. In addition, acetic acid acts as an effective inhibitor that prevents the growth of aerobic spoilage microorganisms, such as yeasts and molds, thereby improving stability against aerobic deterioration after silos are opened for feeding [36]. In addition to the genes necessary to ferment pentoses, the presence of several copies of β-xylosidase genes in *L. hokkaidonensis* (LOOC260_101610, LOOC260_101740, and LOOC260_105960) indicates the ability to utilize xylooligosaccharide.

#### NADPH generation

Unique mechanisms were found for NADPH generation in the *L. vaccinostercus* group LAB. *L. hokkaidonensis*, *L. vaccinostercus*, and *L. suebicus* possessed membrane-bound NAD(P) transhydrogenase PntAB, which mediates the transfer of a hydrogen from NADH to NADP^+^ to produce NADPH using the electrochemical proton gradient [37]. In addition, *L. vaccinostercus* and *L. suebicus* possessed NADP-dependent glyceraldehyde-3-phosphate dehydrogenase, GapN, which catalyzes the one-step conversion of glyceraldehyde-3-phosphate to 3-phosphoglycerate, with the concomitant reduction of NADP^+^ to NADPH [38]. In conventional glycolysis, glyceraldehyde-3-phosphate is converted into 3-phosphoglycerate via a two-step reaction, which is accompanied by the formation of NADH and ATP.

The major cellular source of NADPH is considered to be the oxidative branch of the pentose phosphate pathway, where hexoses are decarboxylated into a C5-moiety. However, pentoses are assimilated without passing through this branch; thus, these enzymes may provide an alternative route for generating NADPH. Analogously, GapN in *Streptococcus mutans*, which lacks the oxidative part of the pentose phosphate pathway, has been suggested to participate in NADPH generation [39]. NADPH mainly functions as an electron donor in anabolic reactions, whereas NAD^+^ mainly functions as an electron acceptor in catabolic reactions. Therefore, both PntAB and GapN are favorable, particularly in the biosynthetic process because they produce a higher NADPH/NADP^+^ ratio and a lower NADH/NAD^+^ ratio.

With the exception of the meat-borne *L. oligofermentans*, the *L. vaccinostercus* group LAB members encode a relatively high number of genes for amino acid biosynthesis. These NADPH generation systems may support the diverse biosynthetic abilities of *L. hokkaidonensis* and its close relatives and may reflect the optimized utilization of pentoses as growth substrates.

## Conclusions

In this study, we successfully reconstructed the complete genome of *L. hokkaidonensis* LOOC260^T^ by whole-genome sequencing using the PacBio SMRT sequencing system and *de novo* assembly based on the HGAP method. We found that the complete genome of *L. hokkaidonensis* LOOC260^T^ contained various previously unreported mobile genetic elements, which included three new types of insertion sequences, two prophage loci, one ICE, and two plasmids, one of which was considered to be a conjugative plasmid. ICE contained many genes related to heavy metal resistance and shared several components with other plant-associated LAB. The ICE may have mediated the dissemination of genes that contributed to niche adaptation in plant-associated LAB species. Our comparative genome analysis also provided insights into the characteristic gene repertoire of this group, such as preferential pentose assimilation. Although our study could not obtain direct evidence of psychrotolerance, we detected possible factors that may contribute to psychrotolerance in this species, such as the uptake of compatible solutes and the synthesis of glutathione. These findings merit further investigations, and the genomic information obtained in this study should facilitate the development of an appropriate silage inoculant for use in cold regions.

## Methods

### Genome sequencing and *de novo* assembly

The cells of *L. hokkaidonensis* LOOC260^T^ were cultured in MRS (de Man, Rogosa, and Sharpe) broth (Difco) and were harvested in the mid-logarithmic phase. The genomic DNA was extracted and purified using Qiagen Genomic-tip 500/G and Qiagen Genomic DNA Buffer Set with lysozyme (Sigma) and proteinase K (Qiagen) according to the manufacturer’s instruction. PacBio SMRT whole-genome sequencing was performed using a PacBio RSII sequencer with P4-C2 chemistry. Four SMRT cells were used for sequencing, thereby yielding 163,376 adapter-trimmed reads (subreads) with an average read length of approximately 4 kbp, which corresponded to approximately 250-fold coverage. *De novo* assembly was conducted using the HGAP method based on the SMRT Analysis package 2.0, which yielded seven contigs. Independent genome sequencing using the 250-bp paired-end Illumina MiSeq system generated 5,942,620 reads, which were assembled into contigs using Platanus assembler ver 1.2 with the default settings [40]. The initial contigs derived from the HGAP method were inspected to determine their continuity with each other based on comparisons with the contigs obtained from the Platanus assembler, and were concatenated into one closed circular chromosome and two circular plasmids. The genome obtained was mapped with reads obtained by the MiSeq system using Burrows-Wheeler Alignment tool (BWA) ver 0.7.5 to detect any assembly and sequence errors [41]. As a result, six one-base-length indels were corrected. The replication origin of the chromosome (*oriC*) was predicted using the Automated Prediction Of Bacterial Replication Origin (APBRO) tool [42], and the chromosome was adjusted so the first base was upstream of the *dnaA* gene in the *oriC* region.

### Plasmid copy number estimation

The plasmid copy numbers were calculated based on the read depth mapped onto each replicon. The reads obtained by the MiSeq system were mapped onto the assembled genome sequences using BWA, and the number of reads mapped onto each replicon was normalized by dividing by its sequence length. The plasmid copy numbers were determined based on the ratio of normalized read numbers for the plasmids relative to that for the chromosome.

### Genome annotation

The genome was annotated using the Microbial Genome Annotation Pipeline (MiGAP) [43] and some of the results were manually curated. In the pipeline, protein coding sequences (CDSs) were predicted by MetaGeneAnnotator 1.0 [44], tRNAs were predicted by tRNAscan-SE 1.23 [45], rRNAs were predicted by RNAmmer 1.2 [46], and functional annotation was finally performed based on homology searches against the RefSeq, TrEMBL, and Clusters of Orthologous Groups (COG) protein databases. Metabolic pathway prediction was performed on KAAS to assign KEGG Orthology (KO) numbers to each predicted CDS [47]. Annotations of the insertion sequences were conducted via the ISsaga web service [48]. Prophage regions were predicted using the PHAge Search Tool (PHAST) web server [49], and its results were confirmed by PCR runs with primers designed to detect phage attachment sites. CRISPR loci were searched for using the CRISPRFinder server [50].

The annotated genome was submitted to the GenomeRefine web service (http://genome.annotation.jp/genomerefine/), which assists with the refinement of annotations and registration at the DNA Data Bank of Japan (DDBJ).

### Comparative genome analysis

The draft genome sequence of *L. suebicus* KCTC 3549^T^ was obtained from GenBank (accession no. BACO01000000). The genomic reads were downloaded from the DDBJ Sequence Read Archive for *L. oligofermentans* DSM 15707^T^, *L. vaccinostercus* DSM 20634^T^, and *L. vaccinostercus* DSM 15802 (accession nos. SRR1151187, SRR1151143, and ERR387466, respectively), which were assembled using the Platanus assembler. These genome sequences were annotated by MiGAP and KAAS in the same manner as *L. hokkaidonensis*. In addition, the genomic data were obtained for 13 representative species in the genus *Lactobacillus* from the NCBI Reference Sequence (RefSeq) database: *Lactobacillus acidophilus* NCFM (NC_006814), *Lactobacillus helveticus* DPC 4571 (NC_010080), *Lactobacillus delbrueckii* subsp. *bulgaricus* ATCC 11842 (NC_008054), *Lactobacillus gasseri* ATCC 33323 (NC_008530), *Lactobacillus reuteri* JCM 1112 (NC_010609), *Lactobacillus fermentum* IFO 3956 (NC_010610), *Lactobacillus buchneri* CD034 (NC_018610, NC_016035, NC_018611, NC_016034), *Lactobacillus brevis* ATCC 367 (NC_008497, NC_008498, NC_008499), *Lactobacillus casei* ATCC 334 (NC_008526, NC_008502), *Lactobacillus rhamnosus* GG (NC_013198), *Lactobacillus plantarum* WCFS1 (NC_004567, NC_006375, NC_006376, NC_006377), *Lactobacillus sakei* subsp. *sakei* 23 K (NC_007576), and *Lactobacillus coryniformis* subsp. *coryniformis* CECT 5711 (NZ_AKFP00000000).

To compare the gene context, all-against-all BLASTP alignments were performed between *L. hokkaidonensis* LOOC260^T^ and each reference strain, and an ortholog table was constructed based on the bidirectional best hit among the BLAST results (Figure 4 and Additional file 2). BLAST alignments were obtained using the following thresholds: cut-off = E-value 0.0001 and ≥30% identity across ≥60% of the sequence length. Each row of the table represented a gene in *L. hokkaidonensis* LOOC260^T^ and its orthologous genes in the reference strains. For each row, the bit scores were divided by the maximum value. Therefore, the numbers in the cells denoted the normalized scores between 0 and 1. Each cell was colored a shade of red according to the normalized score with a deeper color corresponding to a higher score.

### Phylogenetic analysis

A multiple alignment of 16S rRNA nucleotide sequences from 17 species included in the analysis was generated using MUSCLE [51]. The phylogenetic tree was constructed by Mega 5.0 using the neighbor-joining method with a bootstrap value of 1,000 [52].

### Data visualization

The circular genome atlas shown in Figure 1 was produced using Circos software ver 0.66 [53] and in-house python scripts. The linear genome diagrams show in Figure 3 were generated using the GenomeDiagram module in BioPython [54] and they were adjusted manually.

### Availability of supporting data

The complete genome sequence of *L. hokkaidonensis* LOOC260^T^ and its annotations were deposited at DDBJ/ENA/GenBank under accession numbers AP014680 (chromosome), AP014681 (plasmid pLOOC260-1), and AP014682 (plasmid pLOOC260-2). All of the sequencing data were deposited in the DDBJ Sequence Read Archive under accession numbers DRR024500 and DRR024501. The phylogenetic tree and associated data matrix for in Additional file 1: Figure S2 are available in TreeBASE database (Accession URL: http://purl.org/phylo/treebase/phylows/study/TB2:S17206).

## Additional files

Additional file 1: Figure S1. Distributions of species isolated from Timothy grass silage. **Figure S2.** Neighbor-joining tree based on multiple alignments of the 16S rRNA nucleotide sequences from 17 species included in the analysis. **Figure S3.** Reconstructed central carbohydrate metabolism pathway of *L. vaccinostercus* group species. Additional file 2:
**Ortholog table constructed based on the all-against-all BLASTP alignments.** Sheet 1. Each row of the table represents a gene in *L. hokkaidonensis* LOOC260^T^ and its orthologous genes in the reference strains. Each of the values in the cells represents the normalized score divided by the maximum value in the row. Sheet 2. Detailed reports are shown for each BLAST alignment result. Additional file 3:
**Summary of the KAAS annotation results.** Sheet 1. Each cell represents the number of genes assigned to a specific KEGG Orthology (KO) number. Sheet 2. Summarized data for sheet 1. Each cell represents the sum of genes categorized according to a specific metabolic pathway.

## Abbreviations

BLAST: Basic Local Alignment Search Tool
DDBJ: DNA Data Bank of Japan
HGAP: Hierarchical genome assembly process
ICE: Integrated and conjugative element
KAAS: KEGG Automatic Annotation Server
LAB: Lactic acid bacteria
MiGAP: Microbial Genome Annotation Pipeline
SMRT: Single-molecule real-time
