# Supplementary material for: Complete genome sequence and analysis of Lactobacillus hokkaidonensis LOOC260T, a psychrotrophic lactic acid bacterium isolated from silage
Source: BMC Genomics. 2015 Mar 25;16(1):240. doi: 10.1186/s12864-015-1435-2 (PMC4377027; doi:10.1186/s12864-015-1435-2)
Supplement: Additional file 1: Figure S1. — Distributions of species isolated from Timothy grass silage. Figure S2. Neighbor-joining tree based on multiple alignments of the 16S rRNA nucleotide sequences from 17 species included in the analysis. Figure S3. Reconstructed central carbohydrate metabolism pathway of L. vaccinostercus group species. [file 12864_2015_1435_MOESM1_ESM.pdf]

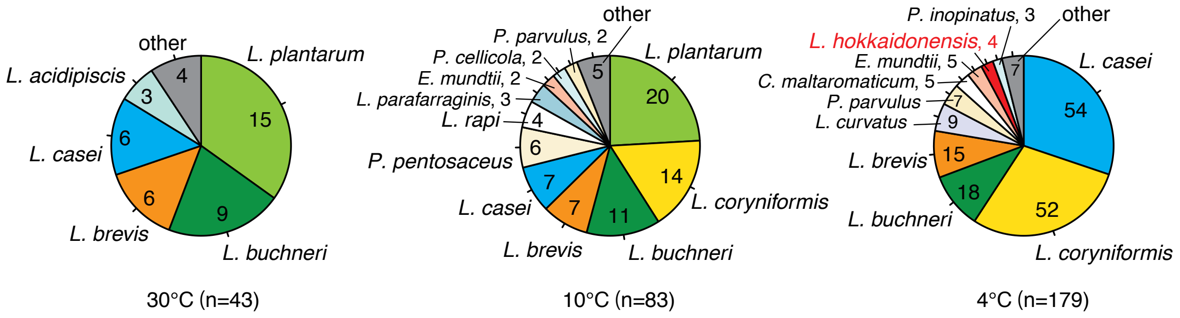

Figure S1. Distributions of species isolated from timothy grass silage stored in Hokkaido in the subarctic northern part of Japan during the winter season. Each strain was isolated after incubation on de Man, Rogosa, and Sharpe (MRS) agar plates for 10 days in anaerobic conditions at three different temperatures: 30°C, 10°C and 4°C. The 16S rRNA gene was sequenced for each isolate using the Sanger sequencing method. Species were identified by querying the sequences against the 16S rRNA sequence database downloaded from NCBI.

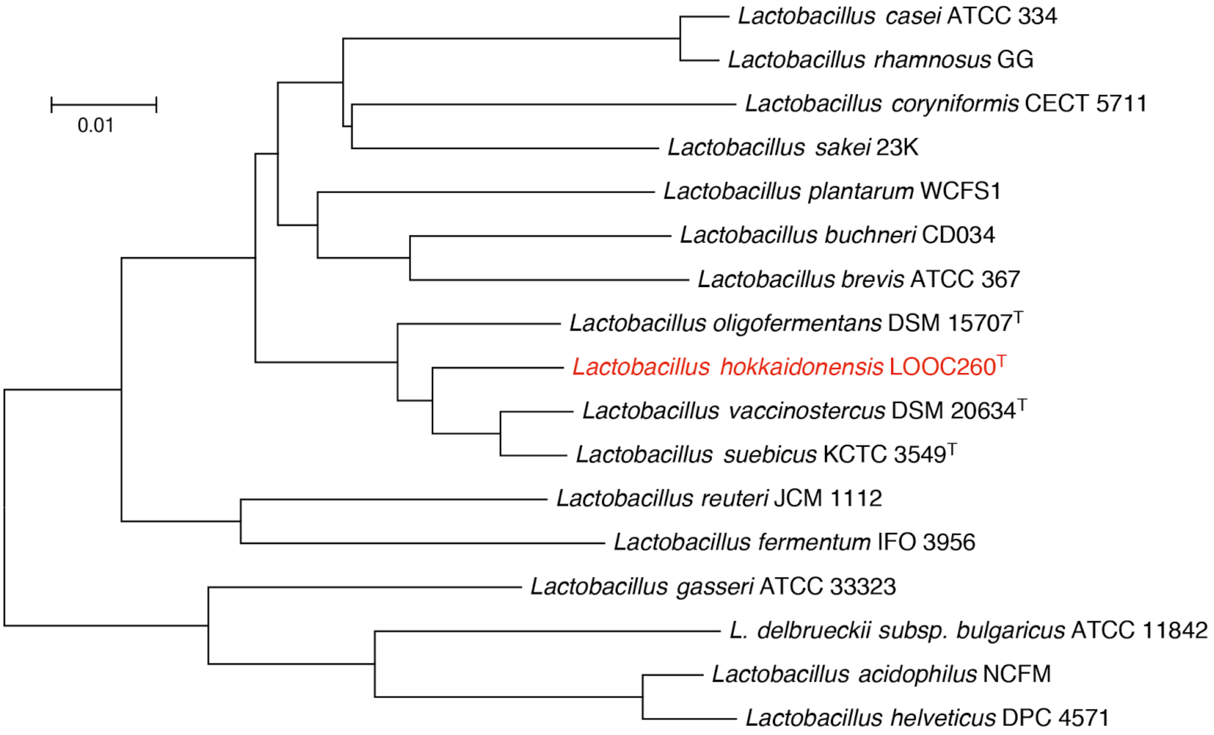

Figure S2. Neighbour-joining tree based on multiple alignment of the 16S rRNA nucleotide sequences from *Lactobacillus hokkaidonensis*, *L. vaccinostercus* group species (*L. vaccinostercus, L. suebicus*, *L. oligofermentans*), and 13 representative species in the genus *Lactobacillus*.

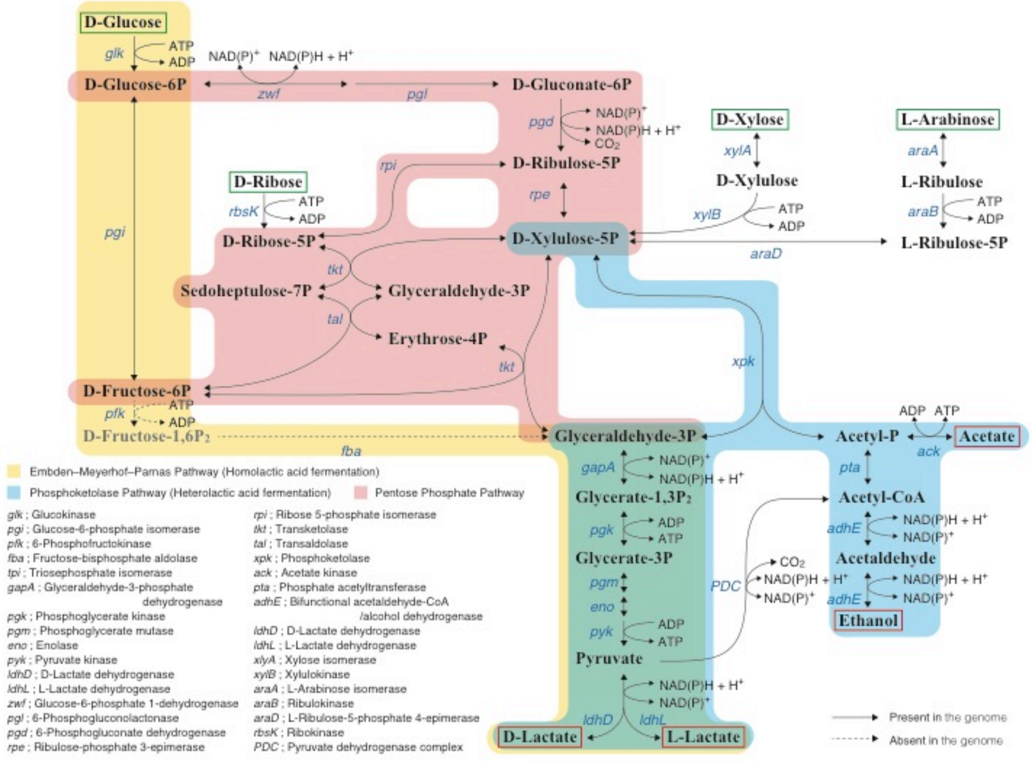

Figure S3. Reconstructed central carbohydrate metabolism pathway of *L. vaccinostercus* group species.
